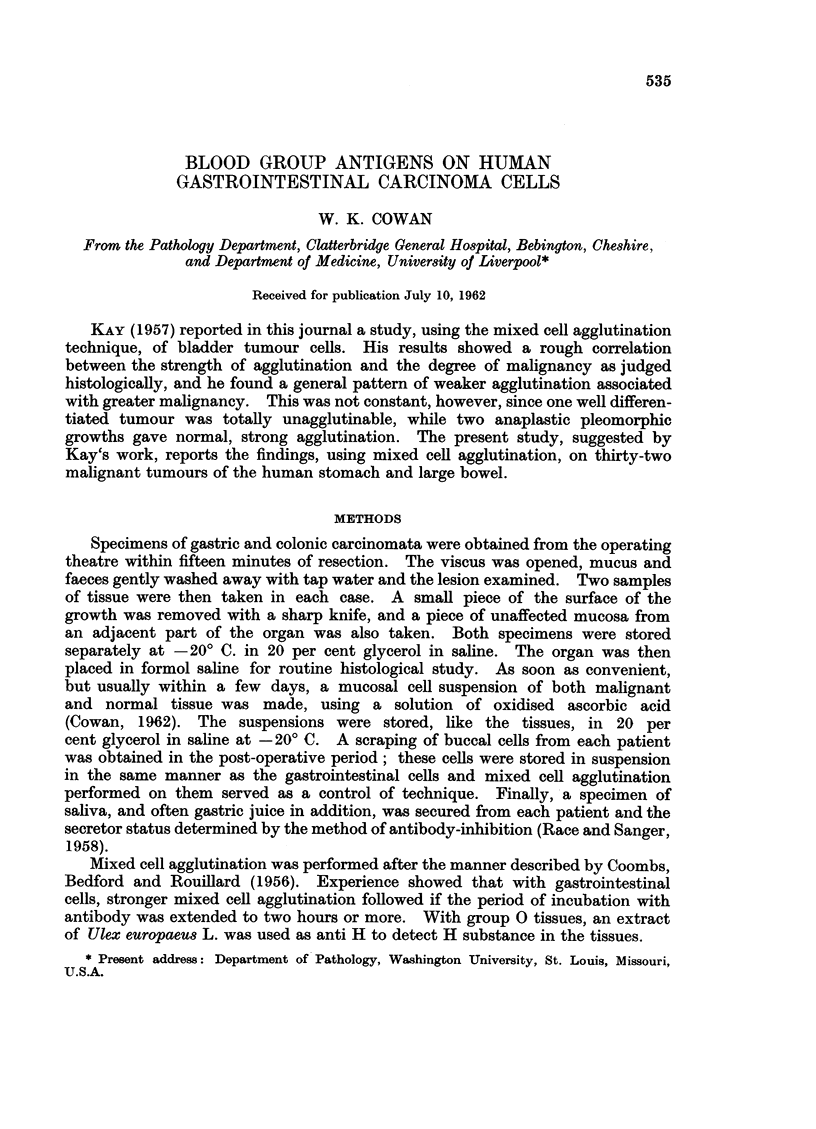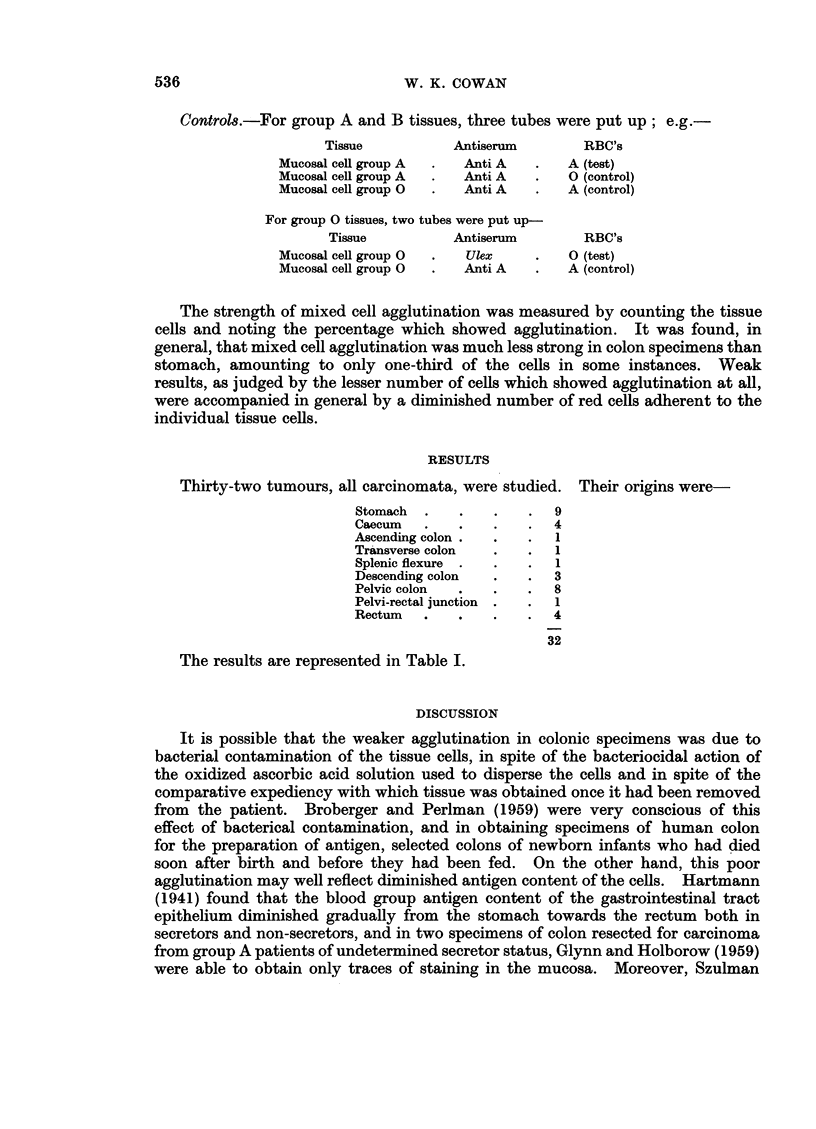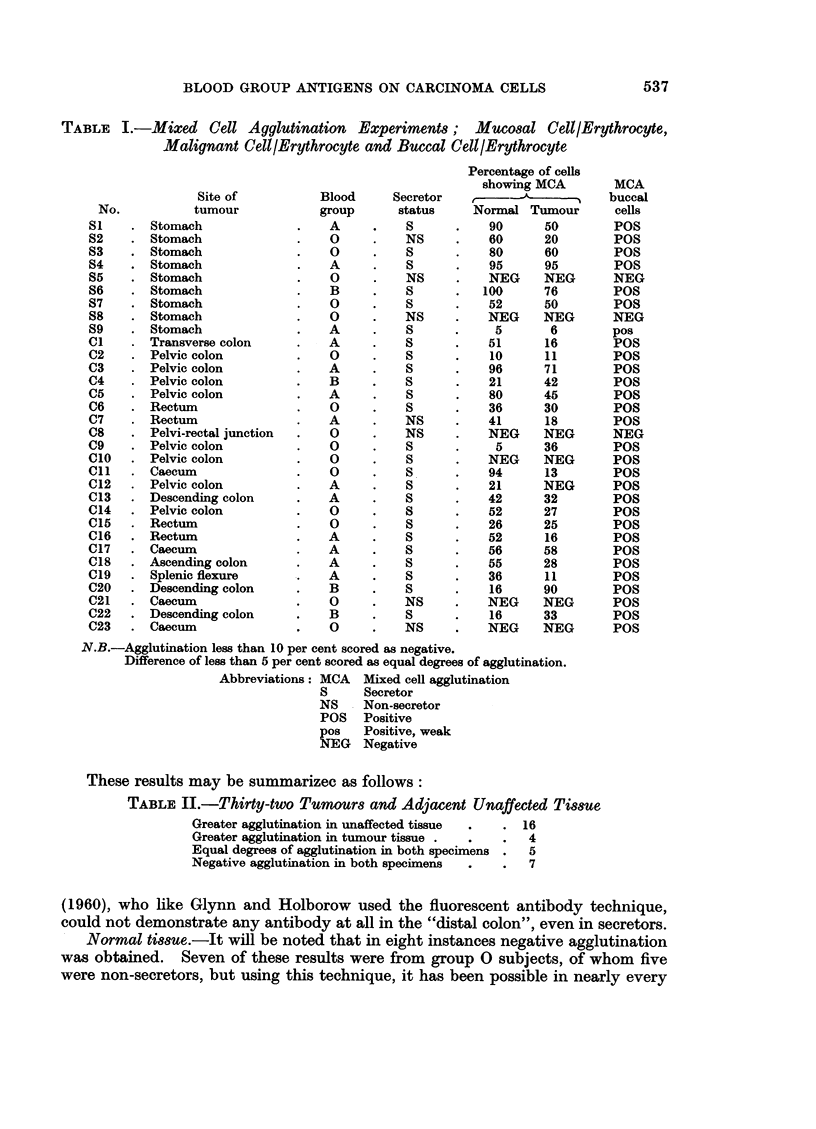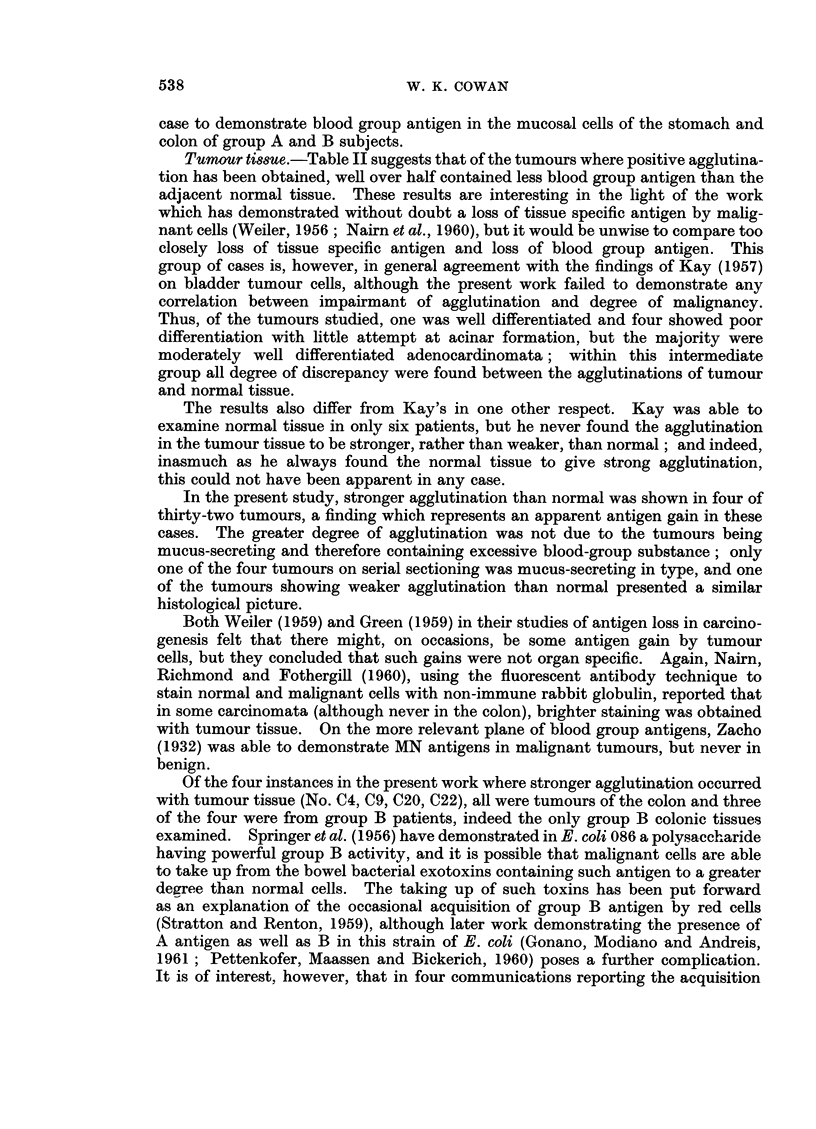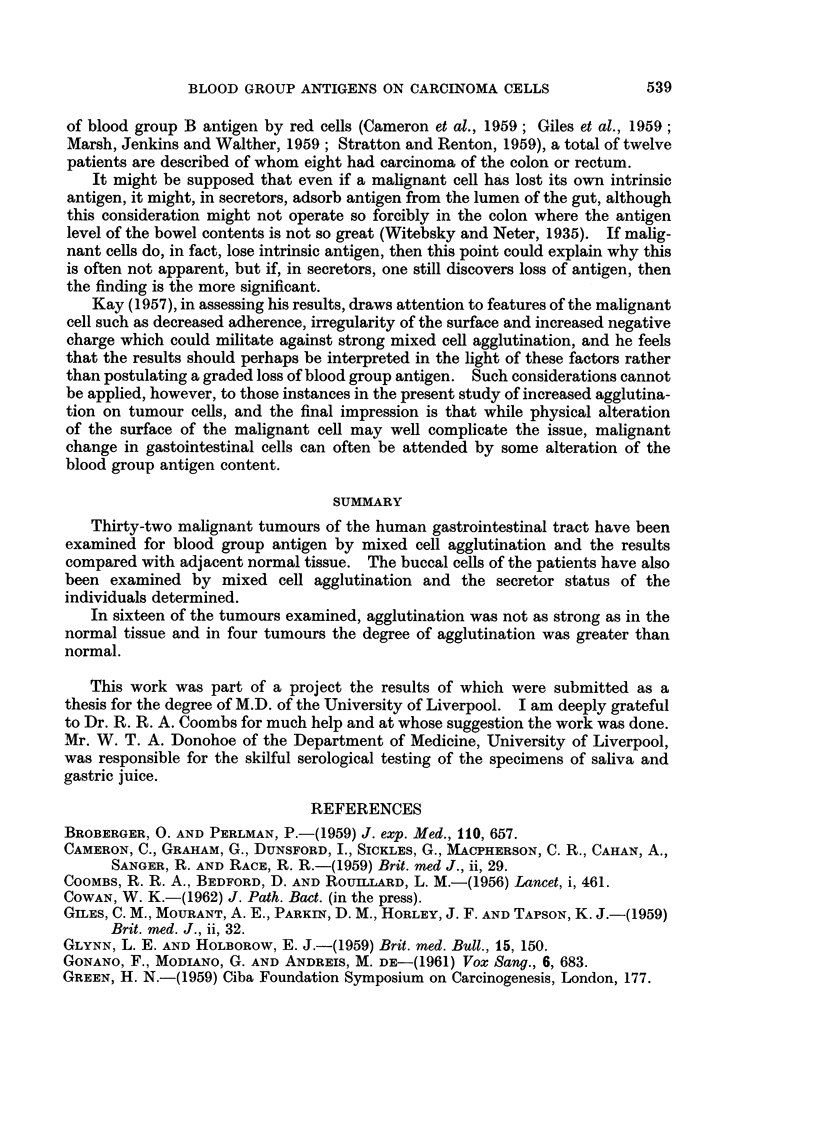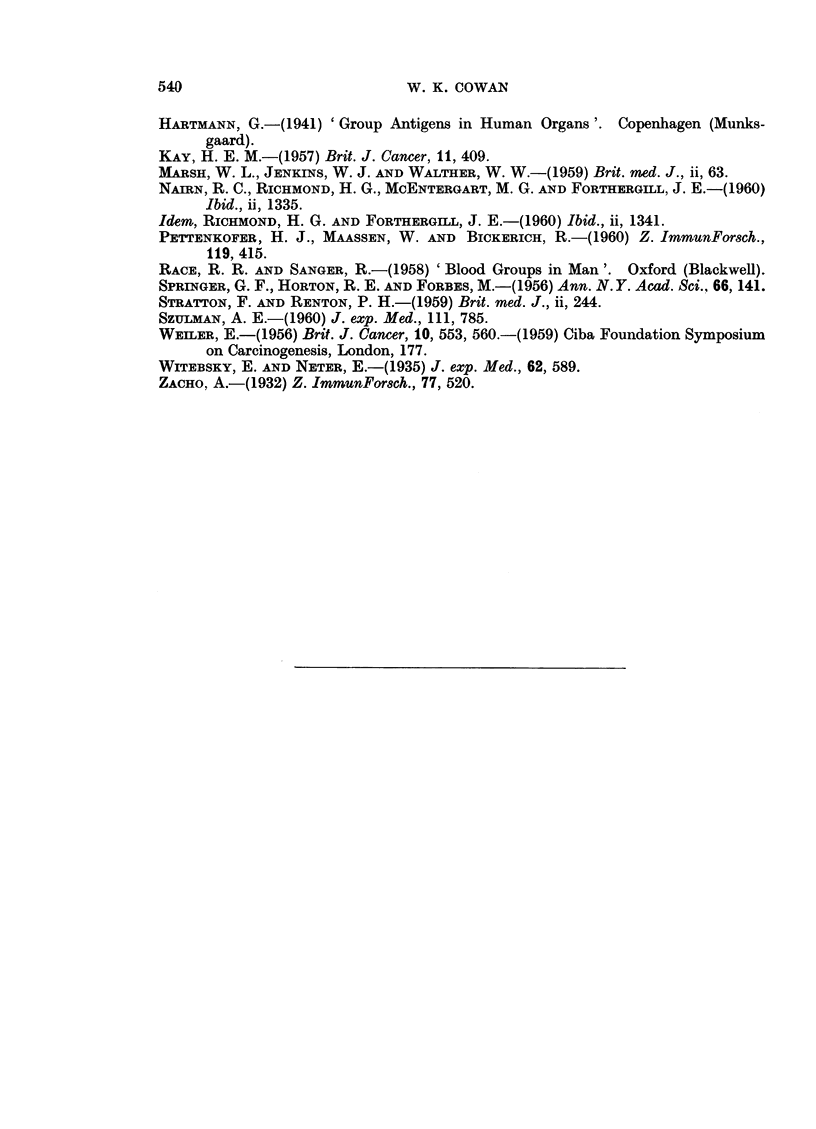# Blood Group Antigens on Human Gastrointestinal Carcinoma Cells

**DOI:** 10.1038/bjc.1962.61

**Published:** 1962-09

**Authors:** W. K. Cowan


### BLOOD GROUP ANTIGENS ON HUMAN GASTROINTESTINAL CARCINOMA CELLS

W. K. COWAN

*From the Pathology Department, Clatterbridge General Hospital, Bebington, Cheshire, and Department of Medicine, University of Liverpool**

Received for publication July 10, 1962

KAY (1957) reported in this journal a study, using the mixed cell agglutination technique, of bladder tumour cells. His results showed a rough correlation between the strength of agglutination and the degree of malignancy as judged histologically, and he found a general pattern of weaker agglutination associated with greater malignancy. This was not constant, however, since one well differentiated tumour was totally unagglutinable, while two anaplastic pleomorphic growths gave normal, strong agglutination. The present study, suggested by Kay's work, reports the findings, using mixed cell agglutination, on thirty-two malignant tumours of the human stomach and large bowel.

### METHODS

Specimens of gastric and colonic carcinomata were obtained from the operating theatre within fifteen minutes of resection. The viscus was opened, mucus and faeces gently washed away with tap water and the lesion examined. Two samples of tissue were then taken in each case. A small piece of the surface of the growth was removed with a sharp knife, and a piece of unaffected mucosa from an adjacent part of the organ was also taken. Both specimens were stored separately at $-20°$ C. in 20 per cent glycerol in saline. The organ was then placed in formol saline for routine histological study. As soon as convenient, but usually within a few days, a mucosal cell suspension of both malignant and normal tissue was made, using a solution of oxidised ascorbic acid (Cowan, 1962). The suspensions were stored, like the tissues, in 20 per cent glycerol in saline at $-20°$ C. A scraping of buccal cells from each patient was obtained in the post-operative period; these cells were stored in suspension in the same manner as the gastrointestinal cells and mixed cell agglutination performed on them served as a control of technique. Finally, a specimen of saliva, and often gastric juice in addition, was secured from each patient and the secretor status determined by the method of antibody-inhibition (Race and Sanger, 1958).

Mixed cell agglutination was performed after the manner described by Coombs, Bedford and Rouillard (1956). Experience showed that with gastrointestinal cells, stronger mixed cell agglutination followed if the period of incubation with antibody was extended to two hours or more. With group O tissues, an extract of *Ulex europaeus* L. was used as anti H to detect H substance in the tissues.

* Present address: Department of Pathology, Washington University, St. Louis, Missouri, U.S.A.

*Controls.*—For group A and B tissues, three tubes were put up ; e.g.—

| Tissue | Antiserum | RBC's |
|---|---|---|
| Mucosal cell group A . | Anti A . | A (test) |
| Mucosal cell group A . | Anti A . | O (control) |
| Mucosal cell group O . | Anti A . | A (control) |

For group O tissues, two tubes were put up—

| Tissue | Antiserum | RBC's |
|---|---|---|
| Mucosal cell group O . | *Ulex* . | O (test) |
| Mucosal cell group O . | Anti A . | A (control) |

The strength of mixed cell agglutination was measured by counting the tissue cells and noting the percentage which showed agglutination. It was found, in general, that mixed cell agglutination was much less strong in colon specimens than stomach, amounting to only one-third of the cells in some instances. Weak results, as judged by the lesser number of cells which showed agglutination at all, were accompanied in general by a diminished number of red cells adherent to the individual tissue cells.

### RESULTS

Thirty-two tumours, all carcinomata, were studied. Their origins were—

| | |
|---|---|
| Stomach . . . . | 9 |
| Caecum . . . . | 4 |
| Ascending colon . . . | 1 |
| Transverse colon . . | 1 |
| Splenic flexure . . . | 1 |
| Descending colon . . | 3 |
| Pelvic colon . . . | 8 |
| Pelvi-rectal junction . . | 1 |
| Rectum . . . . | 4 |
| | 32 |

The results are represented in Table I.

### DISCUSSION

It is possible that the weaker agglutination in colonic specimens was due to bacterial contamination of the tissue cells, in spite of the bacteriocidal action of the oxidized ascorbic acid solution used to disperse the cells and in spite of the comparative expediency with which tissue was obtained once it had been removed from the patient. Broberger and Perlman (1959) were very conscious of this effect of bacterical contamination, and in obtaining specimens of human colon for the preparation of antigen, selected colons of newborn infants who had died soon after birth and before they had been fed. On the other hand, this poor agglutination may well reflect diminished antigen content of the cells. Hartmann (1941) found that the blood group antigen content of the gastrointestinal tract epithelium diminished gradually from the stomach towards the rectum both in secretors and non-secretors, and in two specimens of colon resected for carcinoma from group A patients of undetermined secretor status, Glynn and Holborow (1959) were able to obtain only traces of staining in the mucosa. Moreover, Szulman

TABLE I.—*Mixed Cell Agglutination Experiments; Mucosal Cell/Erythrocyte, Malignant Cell/Erythrocyte and Buccal Cell/Erythrocyte*

| No. | Site of tumour | Blood group | Secretor status | Percentage of cells showing MCA: Normal | Percentage of cells showing MCA: Tumour | MCA buccal cells |
|---|---|---|---|---|---|---|
| S1 | Stomach | A | S | 90 | 50 | POS |
| S2 | Stomach | O | NS | 60 | 20 | POS |
| S3 | Stomach | O | S | 80 | 60 | POS |
| S4 | Stomach | A | S | 95 | 95 | POS |
| S5 | Stomach | O | NS | NEG | NEG | NEG |
| S6 | Stomach | B | S | 100 | 76 | POS |
| S7 | Stomach | O | S | 52 | 50 | POS |
| S8 | Stomach | O | NS | NEG | NEG | NEG |
| S9 | Stomach | A | S | 5 | 6 | pos |
| C1 | Transverse colon | A | S | 51 | 16 | POS |
| C2 | Pelvic colon | O | S | 10 | 11 | POS |
| C3 | Pelvic colon | A | S | 96 | 71 | POS |
| C4 | Pelvic colon | B | S | 21 | 42 | POS |
| C5 | Pelvic colon | A | S | 80 | 45 | POS |
| C6 | Rectum | O | S | 36 | 30 | POS |
| C7 | Rectum | A | NS | 41 | 18 | POS |
| C8 | Pelvi-rectal junction | O | NS | NEG | NEG | NEG |
| C9 | Pelvic colon | O | S | 5 | 36 | POS |
| C10 | Pelvic colon | O | S | NEG | NEG | POS |
| C11 | Caecum | O | S | 94 | 13 | POS |
| C12 | Pelvic colon | A | S | 21 | NEG | POS |
| C13 | Descending colon | A | S | 42 | 32 | POS |
| C14 | Pelvic colon | O | S | 52 | 27 | POS |
| C15 | Rectum | O | S | 26 | 25 | POS |
| C16 | Rectum | A | S | 52 | 16 | POS |
| C17 | Caecum | A | S | 56 | 58 | POS |
| C18 | Ascending colon | A | S | 55 | 28 | POS |
| C19 | Splenic flexure | A | S | 36 | 11 | POS |
| C20 | Descending colon | B | S | 16 | 90 | POS |
| C21 | Caecum | O | NS | NEG | NEG | POS |
| C22 | Descending colon | B | S | 16 | 33 | POS |
| C23 | Caecum | O | NS | NEG | NEG | POS |

*N.B.*—Agglutination less than 10 per cent scored as negative.
Difference of less than 5 per cent scored as equal degrees of agglutination.

Abbreviations:
MCA Mixed cell agglutination
S Secretor
NS Non-secretor
POS Positive
pos Positive, weak
NEG Negative

These results may be summarizec as follows:

TABLE II.—*Thirty-two Tumours and Adjacent Unaffected Tissue*

| | |
|---|---|
| Greater agglutination in unaffected tissue | 16 |
| Greater agglutination in tumour tissue | 4 |
| Equal degrees of agglutination in both specimens | 5 |
| Negative agglutination in both specimens | 7 |

(1960), who like Glynn and Holborow used the fluorescent antibody technique, could not demonstrate any antibody at all in the "distal colon", even in secretors.

*Normal tissue.*—It will be noted that in eight instances negative agglutination was obtained. Seven of these results were from group O subjects, of whom five were non-secretors, but using this technique, it has been possible in nearly every case to demonstrate blood group antigen in the mucosal cells of the stomach and colon of group A and B subjects.

*Tumour tissue.*—Table II suggests that of the tumours where positive agglutination has been obtained, well over half contained less blood group antigen than the adjacent normal tissue. These results are interesting in the light of the work which has demonstrated without doubt a loss of tissue specific antigen by malignant cells (Weiler, 1956 ; Nairn *et al.*, 1960), but it would be unwise to compare too closely loss of tissue specific antigen and loss of blood group antigen. This group of cases is, however, in general agreement with the findings of Kay (1957) on bladder tumour cells, although the present work failed to demonstrate any correlation between impairmant of agglutination and degree of malignancy. Thus, of the tumours studied, one was well differentiated and four showed poor differentiation with little attempt at acinar formation, but the majority were moderately well differentiated adenocardinomata ; within this intermediate group all degree of discrepancy were found between the agglutinations of tumour and normal tissue.

The results also differ from Kay's in one other respect. Kay was able to examine normal tissue in only six patients, but he never found the agglutination in the tumour tissue to be stronger, rather than weaker, than normal ; and indeed, inasmuch as he always found the normal tissue to give strong agglutination, this could not have been apparent in any case.

In the present study, stronger agglutination than normal was shown in four of thirty-two tumours, a finding which represents an apparent antigen gain in these cases. The greater degree of agglutination was not due to the tumours being mucus-secreting and therefore containing excessive blood-group substance ; only one of the four tumours on serial sectioning was mucus-secreting in type, and one of the tumours showing weaker agglutination than normal presented a similar histological picture.

Both Weiler (1959) and Green (1959) in their studies of antigen loss in carcinogenesis felt that there might, on occasions, be some antigen gain by tumour cells, but they concluded that such gains were not organ specific. Again, Nairn, Richmond and Fothergill (1960), using the fluorescent antibody technique to stain normal and malignant cells with non-immune rabbit globulin, reported that in some carcinomata (although never in the colon), brighter staining was obtained with tumour tissue. On the more relevant plane of blood group antigens, Zacho (1932) was able to demonstrate MN antigens in malignant tumours, but never in benign.

Of the four instances in the present work where stronger agglutination occurred with tumour tissue (No. C4, C9, C20, C22), all were tumours of the colon and three of the four were from group B patients, indeed the only group B colonic tissues examined. Springer *et al.* (1956) have demonstrated in *E. coli* 086 a polysaccharide having powerful group B activity, and it is possible that malignant cells are able to take up from the bowel bacterial exotoxins containing such antigen to a greater degree than normal cells. The taking up of such toxins has been put forward as an explanation of the occasional acquisition of group B antigen by red cells (Stratton and Renton, 1959), although later work demonstrating the presence of A antigen as well as B in this strain of *E. coli* (Gonano, Modiano and Andreis, 1961 ; Pettenkofer, Maassen and Bickerich, 1960) poses a further complication. It is of interest, however, that in four communications reporting the acquisition of blood group B antigen by red cells (Cameron *et al.*, 1959 ; Giles *et al.*, 1959 ; Marsh, Jenkins and Walther, 1959 ; Stratton and Renton, 1959), a total of twelve patients are described of whom eight had carcinoma of the colon or rectum.

It might be supposed that even if a malignant cell has lost its own intrinsic antigen, it might, in secretors, adsorb antigen from the lumen of the gut, although this consideration might not operate so forcibly in the colon where the antigen level of the bowel contents is not so great (Witebsky and Neter, 1935). If malignant cells do, in fact, lose intrinsic antigen, then this point could explain why this is often not apparent, but if, in secretors, one still discovers loss of antigen, then the finding is the more significant.

Kay (1957), in assessing his results, draws attention to features of the malignant cell such as decreased adherence, irregularity of the surface and increased negative charge which could militate against strong mixed cell agglutination, and he feels that the results should perhaps be interpreted in the light of these factors rather than postulating a graded loss of blood group antigen. Such considerations cannot be applied, however, to those instances in the present study of increased agglutination on tumour cells, and the final impression is that while physical alteration of the surface of the malignant cell may well complicate the issue, malignant change in gastointestinal cells can often be attended by some alteration of the blood group antigen content.

### SUMMARY

Thirty-two malignant tumours of the human gastrointestinal tract have been examined for blood group antigen by mixed cell agglutination and the results compared with adjacent normal tissue. The buccal cells of the patients have also been examined by mixed cell agglutination and the secretor status of the individuals determined.

In sixteen of the tumours examined, agglutination was not as strong as in the normal tissue and in four tumours the degree of agglutination was greater than normal.

This work was part of a project the results of which were submitted as a thesis for the degree of M.D. of the University of Liverpool. I am deeply grateful to Dr. R. R. A. Coombs for much help and at whose suggestion the work was done. Mr. W. T. A. Donohoe of the Department of Medicine, University of Liverpool, was responsible for the skilful serological testing of the specimens of saliva and gastric juice.

### REFERENCES

Broberger, O. and Perlman, P.—(1959) *J. exp. Med.*, **110**, 657.
Cameron, C., Graham, G., Dunsford, I., Sickles, G., Macpherson, C. R., Cahan, A., Sanger, R. and Race, R. R.—(1959) *Brit. med J.*, ii, 29.
Coombs, R. R. A., Bedford, D. and Rouillard, L. M.—(1956) *Lancet*, i, 461.
Cowan, W. K.—(1962) *J. Path. Bact.* (in the press).
Giles, C. M., Mourant, A. E., Parkin, D. M., Horley, J. F. and Tapson, K. J.—(1959) *Brit. med. J.*, ii, 32.
Glynn, L. E. and Holborow, E. J.—(1959) *Brit. med. Bull.*, **15**, 150.
Gonano, F., Modiano, G. and Andreis, M. de—(1961) *Vox Sang.*, **6**, 683.
Green, H. N.—(1959) Ciba Foundation Symposium on Carcinogenesis, London, 177.

HARTMANN, G.—(1941) ' Group Antigens in Human Organs '. Copenhagen (Munksgaard).
KAY, H. E. M.—(1957) *Brit. J. Cancer*, **11**, 409.
MARSH, W. L., JENKINS, W. J. AND WALTHER, W. W.—(1959) *Brit. med. J.*, ii, 63.
NAIRN, R. C., RICHMOND, H. G., MCENTERGART, M. G. AND FORTHERGILL, J. E.—(1960) *Ibid.*, ii, 1335.
*Idem*, RICHMOND, H. G. AND FORTHERGILL, J. E.—(1960) *Ibid.*, ii, 1341.
PETTENKOFER, H. J., MAASSEN, W. AND BICKERICH, R.—(1960) *Z. ImmunForsch.*, **119**, 415.
RACE, R. R. AND SANGER, R.—(1958) ' Blood Groups in Man '. Oxford (Blackwell).
SPRINGER, G. F., HORTON, R. E. AND FORBES, M.—(1956) *Ann. N.Y. Acad. Sci.*, **66**, **141.**
STRATTON, F. AND RENTON, P. H.—(1959) *Brit. med. J.*, ii, 244.
SZULMAN, A. E.—(1960) *J. exp. Med.*, 111, 785.
WEILER, E.—(1956) *Brit. J. Cancer*, **10**, 553, 560.—(1959) Ciba Foundation Symposium on Carcinogenesis, London, 177.
WITEBSKY, E. AND NETER, E.—(1935) *J. exp. Med.*, **62**, 589.
ZACHO, A.—(1932) *Z. ImmunForsch.*, **77**, 520.